# Unlocking community capabilities across health systems in low- and middle-income countries: lessons learned from research and reflective practice

**DOI:** 10.1186/s12913-016-1859-7

**Published:** 2016-11-15

**Authors:** Asha S. George, Kerry Scott, Eric Sarriot, Barun Kanjilal, David H. Peters

**Affiliations:** 1 School of Public Health, University of the Western Cape, Cape Town, 7535 South Africa; 2 Department of International Health, Johns Hopkins Bloomberg School of Public Health, Baltimore, MD 21205 USA; 3 Global Health Consultant, Bangalore, Karnataka 560084 India; 4 Department of Global Health, Save the Children, Washington DC, 20002 USA; 5 Indian Institute of Health Management Research, Jaipur, 302029 India

The right and responsibility of communities to participate in health service delivery was enshrined in the 1978 Alma Ata declaration [1] and continues to feature centrally in health systems debates today. Communities are a vital part of people-centred health systems [2, 3] and their engagement is critical to realizing the diverse health targets prioritised by the Sustainable Development Goals [4] and the commitments made to Universal Health Coverage [5–7]. Community members’ intimate knowledge of local needs and adaptive capacities are essential in constructively harnessing global transformations related to epidemiological and demographic transitions, urbanization, migration, technological innovation and climate change. Effective community partnerships and governance processes that underpin community capability also strengthen local resilience, enabling communities to better manage shocks, sustain gains, and advocate for their needs through linkages to authorities and services. This is particularly important given how power relations mark broader contexts of resource scarcity and concentration, struggles related to social liberties and other types of ongoing conflicts.

In working with communities, we note that there is a tension between the two extremes of over-stating and under-stating community capabilities. At the over-stating extreme, communities can be seen as having enormous capacity to solve their own problems. This extreme is problematic because it neglects to account for the role of broader contextual factors, such as state failures to provide adequate health services and infrastructure (e.g. roads and sanitation systems) that facilitate health, and broader structural and social determinants driven by colonial history, trade policies, legal systems and political conflicts that deny marginalized groups their rights. Focusing exclusively on community capabilities without considering these contextual factors can lead to solutions that may burden already poor people with unpaid work or financial costs that should be shouldered by the state.

At the under-stating extreme, communities can also be seen as disenfranchised, powerless groups of people or clients, who lack valuable skills or assets and must wait passively for outside help. It fails to recognize that communities have expertise and local resources. They have the right to influence, direct and engage in programs that address their health needs. It also can suggest that communities have little role to play in demanding or supporting improvements in government services—contrary to evidence that social accountability can be an important mechanism for service improvement. Advancing health systems partnerships with communities that are equitable and effective in reaching health goals therefore requires a measured understanding of community capabilities. One that recognizes, harnesses and augments community assets, while also addressing the structural and social determinants of health, whose locus of interventions may lie outside of communities.

As part of its thematic work on ‘Unlocking Community Capabilities’, the Future Health Systems research consortium examines how communities can be active participants in the planning, delivery, monitoring, and evaluation of their health systems, by identifying individual and collective capabilities in diverse social, political, and institutional environments to improve people’s health. This supplement builds on prior work undertaken by the consortium [8, 9] and introduces important concepts through a literature review of key community capability domains in health systems research [10]. It then considers the measurement and effects of community capability on maternal health in Bangladesh, India and Uganda [11], child nutrition in India [12], and care preferences of the elderly in China [13]. Authors also reflect on participatory initiatives building community capability to address maternal health in Uganda [14] and to support health promotion in Bangladesh [15]. Papers also highlight methodological innovations enabling women to dialogue with local decision makers about child health through photovoice [16], and the use of causal loop diagrams to explore scenarios about how trust in vaccination influences health systems and community resilience [17]. A final paper reflects on political philosophy arguments which frame how health systems research can focus on the most vulnerable in low and middle income countries [18].

In the first paper, George et al. [10] describe community capability as the combined influence of a community’s social systems and collective resources that can be applied to address community problems and broaden community opportunities. Drawing from the broad literature on supporting social change among and by communities, the authors posit community capability as consisting of three domains which synergistically support community empowerment: *what communities have; how communities act*, and *for whom do communities act*. These domains encompass material assets and resources, including information, skills and key external linkages that communities must have to support collective endeavours. In addition, it takes into account the governance processes and characteristics that support how assets and resources are shared and controlled; how communities function collectively; and the interests served by the community’s collective action and social processes. Each of these domains is necessary but not sufficient on its own to ensure that communities are empowered to improve their health and well-being. The review found that to date an unfortunate feature of health systems research involving community participation is the limited reporting and low quality of information related to community capability. The authors suggest that having a simpler framework explaining community capability may support better documentation and advance future understanding.

Having started by seeking conceptual clarity about the domains that constitute community capability and their relation to the related concepts of social capital and community competence [19–24], the supplement then engages with the considerable challenge of developing metrics measuring community capability [25]. Composite indexes have measured community capability and evaluated its effects on improving sexual, reproductive, maternal and child health programs [26–28], but standardized, comparable measures can be challenging to establish since capabilities are highly contextualized [11]. Nonetheless, Paina et al. [11], demonstrate the potential quantitative measures of community capability have by finding that the odds of institutional delivery increased by up to 6 % for each capability measured. Barman et al. [12] also found significant effects of perceived community cohesion on the odds of children receiving minimal acceptable diets.

Future research needs to balance ensuring local relevance with comparability across contexts, as well as teasing out more precisely which elements of community capability matter and why. For instance, Barman et al. were surprised to find that membership in credit groups by heads of households and children’s registration at government nutrition and early education centres (*Anganwadi Centres*) were not related to children’s receipt of minimal acceptable diets [12]. Moreover, Liu et al. [13] found that in China, despite the shift in family structure from extended to nuclear families, and the strain on the sibling-less “sandwich generation” supporting their parents and children, most elderly still preferred to stay with their families. Unlike in Western societies, this preference was not influenced by the elderly individual’s health condition. This preference for family-based care in a context of inadequate community support has the potential to exacerbate inequalities by relying on unpaid female labour at home. Future research must continue to evaluate whom in households and communities participate, in what processes, and with what costs to generate better health and well-being.

Health systems research focussed on unlocking community capability entails working with marginalised communities [18]. A key part of that endeavour is building relationships of trust with communities, particularly when prior experiences confronting community vulnerabilities and health systems disruptions have compromised such trust [8, 15, 17]. Regaining trust requires concerted efforts to better comprehend how local contexts inform stakeholder perspectives, before fostering communication and mutual understanding among different community stakeholders. This is shown in the supplement by finding the right communication channels and support for community groups in Uganda [14], using innovative methodologies such as photovoice in India [16], taking the time to participate in social events and ensuring non-affiliation to local political parties in Bangladesh [15] and dialogue between community members and facility providers about their divergent ranking of service quality in Afghanistan [8].

Across the papers, relationship building is a recurrent theme, also used to ensure inclusiveness within communities [10, 14–16]. In Uganda, this involved fostering further communication between family members and linking different types of community actors who were previously not working together [14]. After a year of collaborating and building trust in Bangladesh, data on the lack of female participation in participatory planning processes raised concerns about gender equality, leading to community solutions to address gender inclusion [15]. Even with participatory methodologies such as photovoice, community members who have more time and fewer livelihood concerns were able to participate more than especially marginalised community members [16]. Social inclusion as a part of community capability therefore needs to be continuously assessed rather than assumed.

Ensuring that community interests are placed at the center of health initiatives is one of the reasons why community engagement can be sustained, as has been the case for over 20 years in Bangladesh [15]. However, if community control and ownership is to be respected, initiatives need to be flexible enough to respond to emergent community needs and plans. This may change how activities are undertaken. For example, in Afghanistan, although community meetings were conducted separately for men and women, in one community, women demanded access to the men’s discussions to ensure that their priorities were addressed [8]. Such responsiveness can nonetheless also entail a fine balance in which program implementers struggle with community expectations for tangible financial assistance and concerns about dependence. In Bangladesh, project staff did not provide direct financial resources for activities and avoided taking the lead in community initiatives, instead encouraging community members to take initiative and invite facilitator inputs [15]. However, after years of supporting health promotion without external financial inputs, communities prioritised service delivery and negotiated for financial support to train of local health workers to staff village health posts [15].

The strategic relationship between communities and external actors requires careful mediation [10]. In Uganda, linking community actors upwards to county, sub-county and district health planning modalities was a critical feature of improving maternal health [14]. While the initiative initially only planned to provide a very small travel assistance grant for health volunteers, it ended up providing direct financial support to government health assistants, health workers and community development officers. These government actors were initially unable to provide supervision and support to the village health teams and savings groups (despite being officially mandated to do so) because they had too great a workload and struggled to access transportation or fuel for travel. This highlights how the supportive infrastructure behind building community capability also requires orientation and strengthening.

In conclusion, unlocking community capabilities entails identifying local resources, building capacities, and brokering partnerships. Relationships feature centrally in this. Advancing these capabilities in service delivery models has the potential to advance social equity and strengthen health systems. But there are important nuances and tensions that underpin building and sustaining community capability. Community needs for tangible, material improvements must be met, while also fostering intangible yet powerful elements such as trust. While relationships across diverse actors and levels of the health system need to be brokered, inclusion of the most marginalized people within communities cannot be overlooked. Change needs to be pursued in two directions. Building communities’ internal capabilities needs to be supported, while also strengthening their linkages to external actors, mobilizing resources and supportive mechanisms across health system levels, and the foundations for broader, structural changes.

As much as health systems initiatives seek to work with communities and strengthen their capabilities, communities are diverse and human agency can be ingeniously autonomous. Unlocking community capabilities enables communities to make use of their unique social systems and resources in ways that may not always align with outsider expectations [29]. Policymakers and program implementers must consider what “community participation” means in terms of control over agendas, resources, processes and outcomes as well as in terms of who counts as the community [9].

Engaging with these tensions to strengthen how health systems work with communities demands a careful consideration of how participatory health system interventions at the community level are supported. Interventions must navigate pre-existing power dynamics and the additional tensions created by introducing new resources and expectations. Questioning assumptions about the social processes that underpin how communities participate in health systems interventions is critical to developing more realistic expectations, adequate resources, and supportive principles of collaboration to facilitate community empowerment and broader social development. While there are no magic bullets [30], the papers in this supplement propose new frameworks, generate findings across diverse contexts, provide examples and reflect on key processes that can guide and support the ongoing imperative to unlock community capabilities for health.
